# Brain glucose metabolism in Lewy body dementia: implications for diagnostic criteria

**DOI:** 10.1186/s13195-019-0473-4

**Published:** 2019-02-23

**Authors:** Silvia Paola Caminiti, Arianna Sala, Leonardo Iaccarino, Luca Beretta, Andrea Pilotto, Luigi Gianolli, Sandro Iannaccone, Giuseppe Magnani, Alessandro Padovani, Luigi Ferini-Strambi, Daniela Perani

**Affiliations:** 1 Vita-Salute San Raffaele University, Via Olgettina, 60, Segrate, 20132 Milan, Italy; 2 In Vivo Human Molecular and Structural Neuroimaging Unit, Division of Neuroscience, IRCCS San Raffaele Scientific Institute, Via Olgettina, 60, Segrate, 20132 Milan, Italy; 3 Neurology Unit, Department of Clinical and Experimental Sciences, University of Brescia, Viale Europa, 11, 25123 Brescia, Italy; 4 Parkinson’s Disease Rehabilitation Centre, FERB Onlus S. Isidoro Hospital, Via Ospedale, 34, 24069 Trescore Balneario, Italy; 5 Nuclear Medicine Unit, IRCCS San Raffaele Hospital, Via Olgettina, 60, Segrate, 20132 Milan, Italy; 6 Clinical Neuroscience Department, San Raffaele Turro Hospital, Via Stamira d’Ancona, 20, 20127 Milan, Italy; 7 Department of Neurology and INSPE, San Raffaele Scientific Institute, Via Olgettina, 60, Segrate, 20132 Milan, Italy; 8 Department of Clinical Neurosciences, San Raffaele Scientific Institute, Neurology, Sleep Disorders Center, Via Stamira d’Ancona, 20, 20127 Milan, Italy

**Keywords:** Dementia with Lewy bodies, Brain metabolism, FDG-PET, Biomarker: diagnosis, prognosis

## Abstract

**Background:**

[18F]FDG-PET hypometabolism patterns are indicative of different neurodegenerative conditions, even from the earliest disease phase. This makes [18F]FDG-PET a valuable tool in the diagnostic workup of neurodegenerative diseases. The utility of [18F]FDG-PET in dementia with Lewy bodies (DLB) needs further validation by considering large samples of patients and disease comparisons and applying state-of-the-art statistical methods. Here, we aimed to provide an extensive validation of the [18F]FDG-PET metabolic signatures in supporting DLB diagnosis near the first clinical assessment, which is characterized by high diagnostic uncertainty, at the single-subject level.

**Methods:**

In this retrospective study, we included *N* = 72 patients with heterogeneous *clinical classification at entry* (mild cognitive impairment, atypical parkinsonisms, possible DLB, probable DLB, and other dementias) and an established diagnosis of DLB at a later follow-up. We generated patterns of [18F]FDG-PET hypometabolism in single cases by using a validated voxel-wise analysis (*p* < 0.05, FWE-corrected). The hypometabolism patterns were independently classified by expert raters blinded to any clinical information. The final clinical diagnosis at follow-up (2.94 ± 1.39 [0.34–6.04] years) was considered as the *diagnostic reference* and compared with clinical classification at entry and with [18F]FDG-PET classification alone. In addition, we calculated the diagnostic accuracy of [18F]FDG-PET maps in the differential diagnosis of DLB with Alzheimer’s disease dementia (ADD) (*N* = 60) and Parkinson’s disease (PD) (*N* = 36).

**Results:**

The single-subject [18F]FDG-PET hypometabolism pattern, showing temporo-parietal and occipital involvement, was highly consistent across DLB cases. Clinical classification at entry produced several misclassifications with an agreement of only 61.1% with the diagnostic reference. On the contrary, [18F]FDG-PET hypometabolism maps alone accurately predicted diagnosis of DLB at follow-up (88.9%). The high power of the [18F]FDG-PET hypometabolism signature in predicting the final clinical diagnosis allowed a ≈ 50% increase in accuracy compared to the first clinical assessment alone. Finally, [18F]FDG-PET hypometabolism maps yielded extremely high discriminative power, distinguishing DLB from ADD and PD conditions with an accuracy of > 90%.

**Conclusion:**

The present validation of the diagnostic and prognostic accuracy of the disease-specific brain metabolic signature in DLB at the single-subject level argues for the consideration of [18F]FDG-PET in the early phase of the DLB diagnostic flowchart. The assessment of the [18F]FDG-PET hypometabolism pattern at entry may shorten the diagnostic time, resulting in benefits for treatment options and management of patients.

**Electronic supplementary material:**

The online version of this article (10.1186/s13195-019-0473-4) contains supplementary material, which is available to authorized users.

## Introduction

The diagnosis of dementia with Lewy bodies (DLB) has proven to be challenging, especially in the early disease phase, as the sensitivity changes from 19.4% in early stages to 72.3% in late stages [1]. This might be due to the extremely heterogeneous clinical presentation characterizing early DLB, when core symptoms are not yet evident or partially present. The most frequent DLB misdiagnoses are Alzheimer’s disease (AD), Parkinson’s disease (PD), and other atypical parkinsonisms [1].

Despite all efforts to improve the clinical diagnosis of DLB, further studies are needed to identify a valid biomarker that accurately supports DLB diagnosis even in the early disease phase. [18F]FDG-PET represents a good candidate early biomarker, revealing significant glucose hypometabolism in brain tissues where neuronal death has not yet occurred. Of note, [18F]FDG-PET allows for detection of patterns of brain hypometabolism, the topography of which is specific to different neurodegenerative conditions [2, 3]. In DLB, two decades of research have consistently shown a specific occipital metabolic signature (see Additional file 1: Table S1 for references). Accordingly, [18F]FDG-PET occipital hypometabolism has been long included in the DLB diagnostic criteria [4, 5], although only as a *supportive* biomarker, due to very little evidence supporting its utility for early diagnosis (cfr. [4]). In addition, the suboptimal performance of [18F]FDG-PET biomarker reported so far might be due to the methods used for measuring [18F]FDG-PET hypometabolism [6–8]. For instance, the adoption of a simple visual evaluation of radioactivity distribution, instead of standardized semi-quantitative or quantitative methods, may have some important implications (e.g., [6, 7]). Specifically, the lack of a clear cut-off between a normal and pathological metabolic pattern makes visual assessment difficult and dependent on the observers’ expertise. In the last decade, PET neuroimaging research has focused on the development of tools to provide a reliable definition of hypometabolism patterns. The processing of [18F]FDG-PET data by means of validated semi-quantitative methods is strongly recommended by the international scientific societies to improve diagnostic accuracy in neurodegenerative conditions [7–12]. Thus, the ambiguity associated with visual assessment and the  general support for semi-quantitative methods of [18F]FDG-PET data processing argues for the development of more effective parametric/quantitative approaches to assess [18F]FDG-PET in DLB diagnosis. We thus considered a large sample of patients with a diagnosis of probable DLB, as evaluated at follow-up. We assessed, at the single-subject level, the alterations in brain glucose metabolism characterizing this cohort of patients using standardized t-maps of hypometabolism. The aim of this retrospective study was to provide an extensive evaluation of the patterns of brain hypometabolism in a large sample of cases diagnosed with probable DLB at follow-up, in order to test [18F]FDG-PET accuracy (i) in predicting the final clinical diagnosis at a long-term follow-up and (ii) in the differential diagnosis of DLB with its most frequent misdiagnoses, namely AD dementia (ADD) and PD [1].

## Materials and methods

### Study design

From a large clinical cohort referred to the Departments of Neurology and Nuclear Medicine Unit of San Raffaele Hospital (Milan, Italy) between 2010 and 2015, we retrospectively selected 72 patients who had a final clinical diagnosis of probable DLB. Each patient received a clinical assessment at entry, defined in this study as “clinical classification at entry”. Notably, at the time of the first clinical assessment, they were characterized by ambiguous and heterogeneous clinical presentations, making the clinical diagnosis challenging. Within 3 months of the first clinical assessment, an [18F]FDG-PET scan was obtained for each patient, as is standard procedure at San Raffaele Hospital. After a mean follow-up period of 2.94 ± 1.39 years, a final clinical diagnosis was made in accordance with DLB diagnostic criteria (“diagnostic reference”) [5].

We assessed the value of [18F]FDG-PET classification alone (i) in the predictive diagnostic performance and (ii) in the discriminative diagnostic performance.

### Participants

**Fig. 1:**
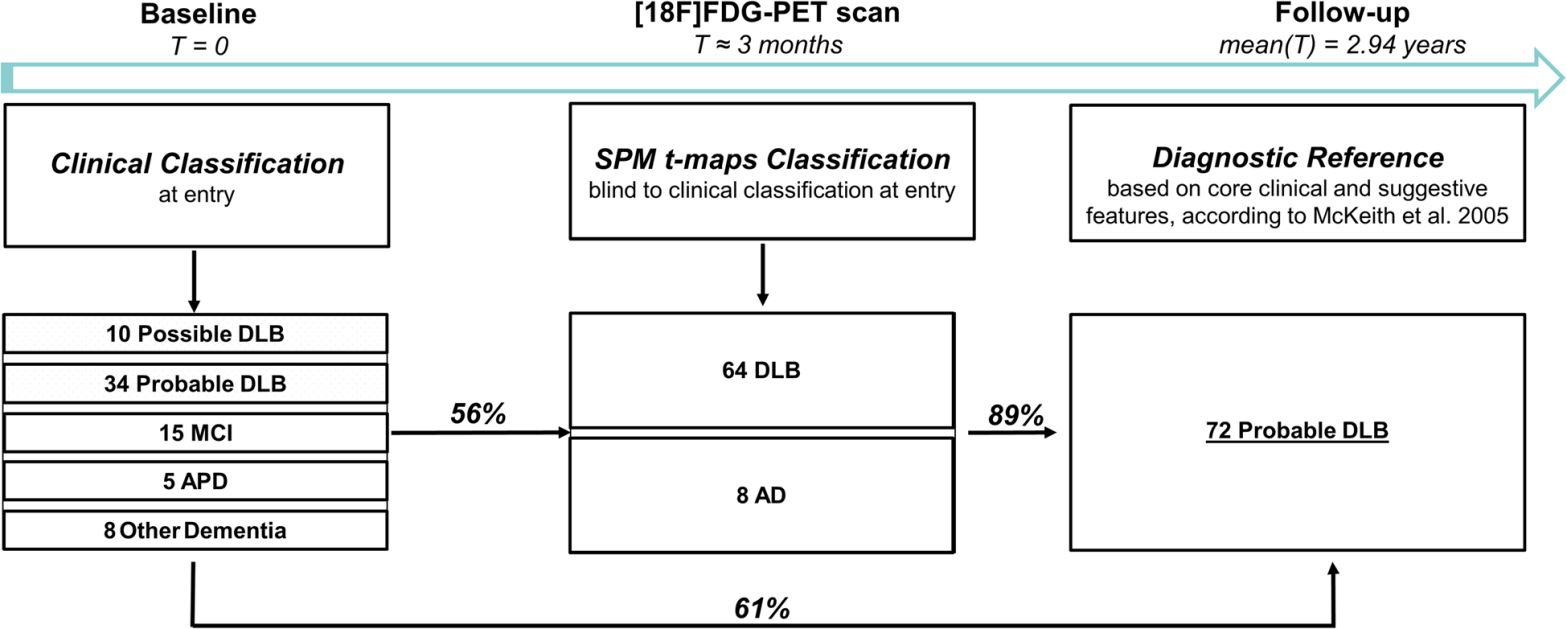
Flowchart of the classification process of the 72 DLB patients. Flowchart depicts the classifications of patients according to clinical classification at entry (*T* = 0), [18F]FDG-PET SPM classification (*T* ≈ 3 months) and final clinical diagnosis at follow-up (*T* ≈ (2.94 ± 1.39 [0.34–6.04] years). Abbreviations: ADD, Alzheimer’s disease dementia; APD, atypical parkinsonian disorders; DLB, dementia with Lewy bodies; MCI, mild cognitive impairment; PD, Parkinson’s disease

At the time of the first clinical assessment, *N* = 34 patients were classified as probable DLB, *N* = 10 as possible DLB [5], *N* = 5 as atypical parkinsonism (unspecified subtypes), *N* = 15 as mild cognitive impairment (MCI) [13], and *N* = 8 as another dementia (i.e., four suspected probable ADD, one fronto-temporal dementia, and three dementia with uncertain diagnosis) (see Fig. 1; Table 1). In possible DLB cases, there was very doubtful presence of visual hallucinations or cognitive fluctuations, only reported by the relatives during the interviews but not tested through specific psychometric assessments (e.g., [14, 15]) or evaluated by standard sequential neuropsychological exams.

**Table 1:** Demographics and clinical features of the case series at entry and follow-up

*Clinical classification at entry*	Possible DLB	Probable DLB	Atypical parkinsonism	MCI	Other dementias
*N*	10	34	5	15	8
Gender (M/F)	4/6	21/13	3/2	10/5	4/4
Age at PET scan (years)	70.40 ± 7.93 (59–81)	74.56 ± 7.94 (48–93)	68.20 ± 8.08 (57–77)	68.80 ± 5.47 (58–78)	75.00 ± 6.14 (68–88)
MMSE score at entry	19.11 ± 3.68 (14–24)	17.87 ± 5.21 (7.70–26.70)	18.76 ± 2–20 (15.60–20.30)	22.20 ± 2.86 (18.9–27)	13.36 ± 2.06 (10–15.3)
Disease duration at entry (years)	3.68 ± 2.75 (1.00–10.75)	2.38 ± 1.53 (0.20–6.00)	2.30 ± 2.09 (0.30–5.50)	1.23 ± 0.83 (0.33–2.90)	3.59 ± 2.07 (0.20–7.00)
Time of follow-up (years)	2.70 ± 1.63 (0.34–4.36)	3.09 ± 1.46 (0.77–6.04)	3.71 ± 1.29 (2.92–5.20)	2.89 ± 0.32 (0.78–4.93)	1.41 ± 0.39 (1.16–1.86)
Visual hallucinations (%)	30%	53%	40%	53%	63%
Cognitive fluctuations (%)	0%	35%	20%	27%	38%
Parkinsonism (%)	100%	94%	100%	67%	75%
^a^Clinical classification at follow-up	Probable DLB	Probable DLB	Probable DLB	Probable DLB	Probable DLB
Visual hallucinations (%)	90%	74%	80%	67%	88%
Cognitive fluctuations (%)	70%	82%	60%	53%	63%
Parkinsonism (%)	100%	100%	100%	80%	75%
RBD (%)	40%	62%	20%	40%	38%
Neuroleptic sensitivity (%)	10%	6%	0%	0%	0%
Available DATSCAN (N)	3 out of 10 (positive)	7 out of 34 (positive)	3 out of 5 (positive)	1 out of 15 (positive)	0 out of 8

^a^Clinical Research Criteria for probable DLB diagnosis according to McKeith et al. 2005

Data are reported as mean ± standard deviation (range)

*Abbreviations*: *DLB* dementia with Lewy bodies, *MCI* mild cognitive impairment

Expert neurologists made the final diagnosis of probable DLB at follow-up in the presence of at least two core symptoms (i.e., cognitive fluctuations, visual hallucination (VH), or parkinsonism), or one core symptom plus at least one suggestive feature (i.e., RBD, severe neuroleptic sensitivity, or low dopamine transporter uptake at SPECT) [5]. Specifically, presence of parkinsonism was defined as the presence of one or more spontaneous cardinal features, namely bradykinesia (defined as slowness of movement and decrement in amplitude or speed), rest tremor, or rigidity. Cognitive fluctuations were defined as alternating periods of cognitive impairment and normal or near-normal cognitive function also accompanied by pronounced variations in attention and alertness, according to serial cognitive examinations and the detailed anecdotal interviews with patients and caregivers. VH were considered present when patients experienced well-formed images of people, animals, or objects, as reported by detailed anecdotal interviews with patients and caregivers. Diagnosis of RBD was made according to the international diagnostic criteria (American Academy of Sleep Medicine, 2014).

In addition, in some cases, supportive biomarkers were also employed to aid the final diagnosis (“diagnostic reference”), namely [18F]FDG-PET (100%, positivity 89%), CT/MRI (100%, positivity 68%), and EEG (51%, positivity 46%). The percentage of presence of DLB core clinical features and supportive features (DaTSCAN and RBD) in probable DLB cases at follow-up is available in Table 1 [5].

Cognitive functions were evaluated by a team of neuropsychologists by means of a thorough neuropsychological battery. The disease duration was defined as the time between the appearance of the earliest clinical symptoms, as reported by the patients and/or caregiver, and the clinical assessment at entry. Demographics at entry are reported in Table 1.

In order to provide a thorough sample of cases for the most challenging differential diagnoses of DLB defined previously, we included two additional patient groups: ADD (age in years 68.87 ± 6.28; MMSE 19.16 ± 4.85; disease duration in years 3.07 ± 1.69) and PD (age in years 62.47 ± 10.91; MMSE 28.76 ± 1.23; disease duration in years 4.75 ± 2.57). Both groups were part of the internal clinical and PET imaging databases from the participating centers (the Neurology Departments and Nuclear Medicine Unit of San Raffaele Hospital, Milan; Neurology Unit, Department of Clinical and Experimental Sciences, University of Brescia, Brescia, Italy). In detail, we included 60 patients diagnosed as probable ADD, in accordance with standard diagnostic criteria [16]. Diagnosis of probable ADD was confirmed in all cases at follow-up (3.25 ± 1.05 years) and supported by evidence of AD pathophysiological processes [16], as shown by either cerebrospinal fluid (available in 67% cases), MRI (65%), and/or [18F]FDG-PET exams (100%). The group was comprised of typical ADD cases and five patients with the posterior cortical atrophy (PCA) variant, a number consistent with the estimated 5–10% prevalence of ADD-PCA in ADD [17]. We also included 36 patients diagnosed as idiopathic PD in accordance with standard diagnostic criteria [18], with a clinical follow-up (4.02 ± 0.2 years) and no dementia. The diagnosis of PD was clinically confirmed according to the neurological examination at follow-up and sustained response to levodopa treatment.

### [18F]FDG-PET

[18F]FDG-PET scans were acquired using a Discovery STE PET scanner (3.27 mm thickness; 5.55 mm in-plane FWHM), manufactured by GE Healthcare. The [18F]FDG-PET acquisition procedures conformed to the European Association of Nuclear Medicine guidelines [19]. Static emission images were acquired 45 min after injecting 185–250 MBq of [18F]FDG via a venous cannula, with 15-min scan duration. Data obtained from steady-state static [18F]FDG-PET acquisition were demonstrated to be comparable to the [18F]FDG-PET data obtained from dynamic quantitative acquisition procedures [20].

All images were reconstructed using an ordered subset-expectation maximization algorithm. Attenuation correction was based on CT scans. Each reconstructed image was visually inspected to check for major artifacts. Image pre-processing was performed using SPM5 software (http://www.fil.ion.ucl.ac.uk/spm/software/ spm5/), running in Matlab (MathWorks Inc., Sherborn, MA, USA. Images were spatially smoothed with an isotropic 3D Gaussian kernel (FWHM 8–8–8 mm). Global mean scaling was applied to each image in order to account for between-subject uptake variability [21].

### Statistical analysis

#### Group analysis

We performed a voxel-wise group analysis to obtain the common pattern of brain hypometabolism in the final DLB group, namely in those patients with a DLB diagnosis confirmed at follow-up. We obtained the common hypometabolism pattern by comparing the DLB group with a large database of normal controls (*N* = 112; age 64.68 ± 9.35; M/F 53/59) by means of a *t*-test statistical comparison on SPM, entering age as nuisance covariate. Extensive information on the characteristics of the normal control case series can be found elsewhere [6, 22]. The statistical threshold was set at *p* = 0.05, FWE-corrected for multiple comparisons. Only clusters containing more than 100 voxels were deemed to be significant.

#### Optimized single-subject [18F]FDG-PET SPM analyses

Individual patient hypometabolism SPM-t-maps were obtained by means of a two-sample *t*-test with comparison to a large database of 112 normal controls and with age entered as a nuisance covariate (*p* < 0.05 FWE-corrected, minimum cluster extent *k* = 100 voxels). SPM-t-maps were evaluated by four imaging experts, who classified patterns of brain hypometabolism according to the disease-specific patterns reported in the literature [4, 6, 23, 24]. In order to provide an unbiased [18F]FDG-PET classification, each rater was blinded to the SPM-t-maps classifications produced by the other experts, the clinical diagnoses, and any other information about the patient. Patients received a hypometabolism classification based on their topographical features. Medial and lateral occipital cortex hypometabolism with involvement of temporo-parietal and frontal cortices represents the DLB-like metabolic pattern [4, 23], bilateral temporo-parietal and/or precuneus/posterior cingulate cortex hypometabolism represents the AD-like metabolic pattern [6], temporo-parietal and occipital hypometabolism, associated with hypometabolic foci in the frontal eye field regions, represents the hypometabolism pattern in PCA variant of AD [25], and very limited brain hypometabolism confined to premotor and motor regions is the typical pattern in PD [24, 26].

Inter-rater agreement was assessed by Cohen’s *K*. The SPM-t-map classification was computed based on a supermajority criterion.

The *predictive diagnostic performance* of the [18F]FDG-PET single-subject SPM-t-map classification was evaluated by computing the agreement between SPM-t-map classification and the diagnostic reference. We also computed the agreement between classification at entry and diagnostic reference (McNemar test).

The *discriminative diagnostic performance* of the [18F]FDG-PET single-subject SPM-t-maps was evaluated by using measures of sensitivity, specificity, and accuracy, considering the final clinical diagnosis at follow-up as the diagnostic reference. We selected a series of features as hallmarks for discriminating DLB from the other conditions, namely (i) the presence/absence of hypometabolism in the occipital lobe, (ii) the cingulate island sign (CIS) [27], (iii) the presence/absence of hypometabolism in dorsolateral prefrontal cortex (DLPFC), more prominent in DLB as compared to ADD-PCA patients [25], and (iv) hypometabolism asymmetry, more prevalent in ADD-PCA [28]. The cingulate island sign was quantified as previously described [27] by calculating the ratio between [18F]FDG-PET uptake in the posterior cingulate cortex and the sum of the [18F]FDG-PET uptake in precuneus and cuneus regions [27]. A ROC analysis was then run to find the optimal cut-off CIS value to discriminate between DLB and ADD subjects. Performance of hypometabolism hallmarks was evaluated as above and directly compared in a ROC curve analysis by means of a DeLong test for paired samples.

#### Hierarchical cluster analysis

In order to investigate the presence of metabolic subtypes within our patient series, we performed a hierarchical cluster analysis on regional [18F]FDG-PET hypometabolism (Table 2). To do so, we selected a set of *N* = 14 a priori defined regions of interest (ROIs) known to be involved in dementia with Lewy bodies [23, 29] and extracted regional hypometabolism values from the single-subject SPM-t-maps. These ROIs were derived from (i) the Automated Anatomical Labelling (AAL) Atlas [30] for the calcarine cortex, cuneus, superior occipital gyrus, middle occipital gyrus, inferior occipital gyrus, fusiform gyrus, lingual gyrus, inferior temporal gyrus, middle temporal gyrus, superior temporal gyrus, and precuneus, (ii) Sallet’s Dorsal Frontal Parcellation Atlas [29] for the DLPFC, and (iii) the AD-related MetaROIs as proposed by Landau and colleagues [30] for the posterior cingulate cortex and angular gyri.

**Table 2:** Clinical and neuropsychological features of the case series at entry subdivided according with the hierarchical cluster analysis into subgroup A and subgroup B (see text)

	Subgroup A	Subgroup B	Sign.
Visual hallucinations (%)	43.9%	58.1%	.234
Cognitive fluctuations (%)	26.8%	29.0%	.836
Parkinsonism (%)	85.4%	90.3%	.529
Disease duration (mean ± sd)	2.43 ± 2.13	2.52 ± 1.53	0.835
MMSE (mean ± sd)	19.96 ± 4.39	16.24 ± 4.87	0.003^*^
Attentive matrices (mean ± sd)	28.05 ± 12.75	16.29 ± 12.03	0.003*
Clock drawing test (mean ± sd)	2.28 ± 2.95	0.37 ± 1.16	0.013*
Corsi span forward (mean ± sd)	2.02 ± 1.89	2.29 ± 1.59	0.673
Digit span forward (mean ± sd)	4.83 ± 0.99	4.38 ± 1.64	0.23
Semantic fluency (mean ± sd)	22.05 ± 9.47	20.06 ± 9.48	0.462
Phonemic fluency (mean ± sd)	16.03 ± 11.08	12.71 ± 11.47	0.302
Raven’s Colored Progressive Matrices (mean ± sd)	19.17 ± 6.74	13.61 ± 9.65	0.04*
Rey figure—copy (mean ± sd)	11.53 ± 10.77	9.77 ± 9.71	0.623
Rey figure—delayed recall (mean ± sd)	4.63 ± 4.12	4.33 ± 4.16	0.84
Rey Verbal Learning Test (mean ± sd)	2.46 ± 2.95	1.72 ± 2.75	0.496
Short story—delayed recall (mean ± sd)	5.2 ± 3.42	4.69 ± 5.66	0.727

Data at baseline

*Abbreviation*: *sd* standard deviation; *significant at *p*<0.05 at two-sample t-test

The obtained ROIs × subjects matrix was considered for the principal component analysis that was performed to reduce dimensionality. Individual scores then entered a hierarchical clustering analysis based on maximum distances (*supremum norm*) with the Ward clustering method. We had no a priori assumptions on an optimal cluster solution. After selection of the optimal cluster solution, the subgroup-specific patterns of [18F]FDG-PET hypometabolism were evaluated both by means of an age-corrected two-sample *t*-test performed in SPM and an evaluation of the regional hypometabolism at the single-subject level, as described above. Subgroup differences in symptom prevalence and severity of neuropsychological deficits were tested by means of Pearson’s chi-square tests and multiple independent sample *t*-tests, respectively. All the analyses were performed with RStudio software built-in packages (v. 1.0.136) [31] and FactoMineR package [32].

## Results

**Fig. 2:**
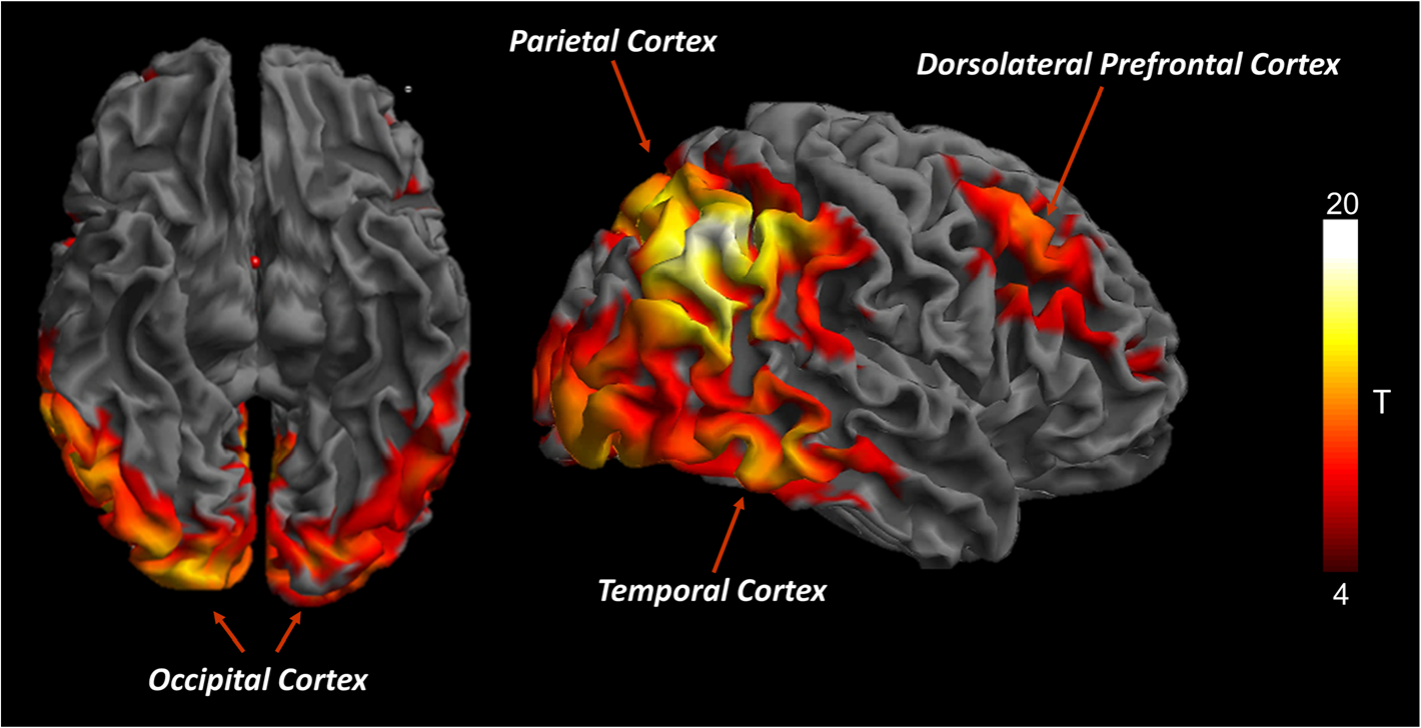
Topographical distribution of brain hypometabolism in the whole DLB group. [18F]FDG-PET SPM group analysis obtained by statistically comparing DLB group with healthy controls database (see text for details). Yellow/red scales represent hypometabolism severity (*p* < 0.05 FWE-corrected, *k* = 100)

**Fig. 3:**
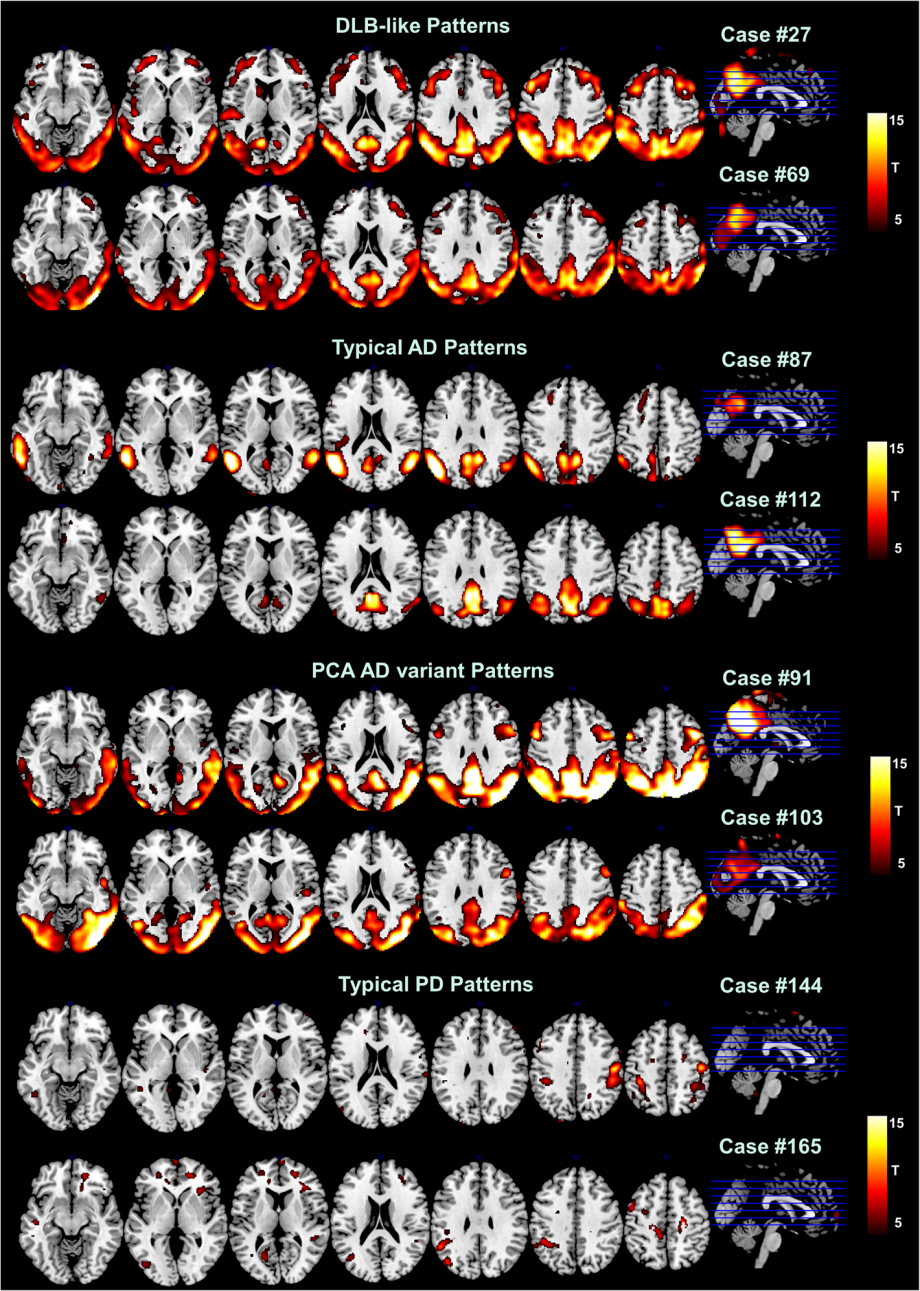
Representative SPM-t-map at the single-subject level. Figure shows examples of [18F]FDG-PET single-subject SPM-t-maps across neurodegenerative conditions. Yellow/red scales represent hypometabolism severity, as obtained from the comparison with the normal control database (*p* < 0.05 FWE-corrected, *k* = 100). Abbreviations: AD, Alzheimer’s disease; DLB, dementia with Lewy bodies; PCA, posterior cortical atrophy; PD, Parkinson’s disease

For the brain hypometabolism assessment, the common DLB hypometabolism pattern is presented in Fig. 2. The DLB hypometabolism pattern obtained at the group level involved the lateral and medial occipital cortex, temporo-parietal cortex, and frontal cortex. This topographical distribution was consistently found in the hypometabolism SPM-t-maps obtained at the single-subject level with our optimized SPM procedure (see Fig. 3 for illustrative examples).

The independent raters identified the DLB-like pattern in 64 out of 72 cases clinically diagnosed as probable DLB at follow-up, 3 out of 60 ADD cases, and 5 out of 36 PD cases. The AD-like pattern was identified in 8 out of 72 DLB cases and 57 out of 60 ADD cases. The PD-like pattern was reported in 31 out of 36 clinically diagnosed PD and was not reported in other conditions (Table 3). The SPM-t-map rater classification within the whole sample of 168 cases yielded substantial inter-rater agreement (Cohen’s *K* mean 0.80).

**Table 3:** Prevalence and performance of hypometabolism patterns in discriminating DLB from ADD and PD patients

Prevalence (%) of hypometabolism patterns			
	DLB-like	AD-like	PD-like
DLB	64/72 (88.9%)	8/72 (11.1%)	0/72 (0%)
ADD	3/60 (5%)	57/60 (95%)	0/60 (0%)
PD	5/36 (13.8%)	0/36 (0%)	31/36 (86.1%)
Discriminative performance of hypometabolism patterns			
	Sensitivity	Specificity	Accuracy
DLB vs. ADD	0.89 (0.79–0.95)	0.95 (0.86–0.99)	0.92 (0.86–0.96)
DLB vs. PD	1 (0.94–1)	0.86 (0.70–0.95)	0.95 (0.89–0.98)

*Abbreviations*: *ADD* Alzheimer’s disease dementia, *DLB* dementia with Lewy bodies, *PD* Parkinson’s disease

The predictive diagnostic performance of [18F]FDG-PET single-subject SPM-t-maps yielded 88.89% agreement with the final clinical diagnosis at follow-up (diagnostic reference), with a significant increase (45.5%) of correct classification as compared to the clinical classification at entry alone (*χ*^2^ = 12.03, *p* < 0.001) (Fig. 1).

**Fig. 4:**
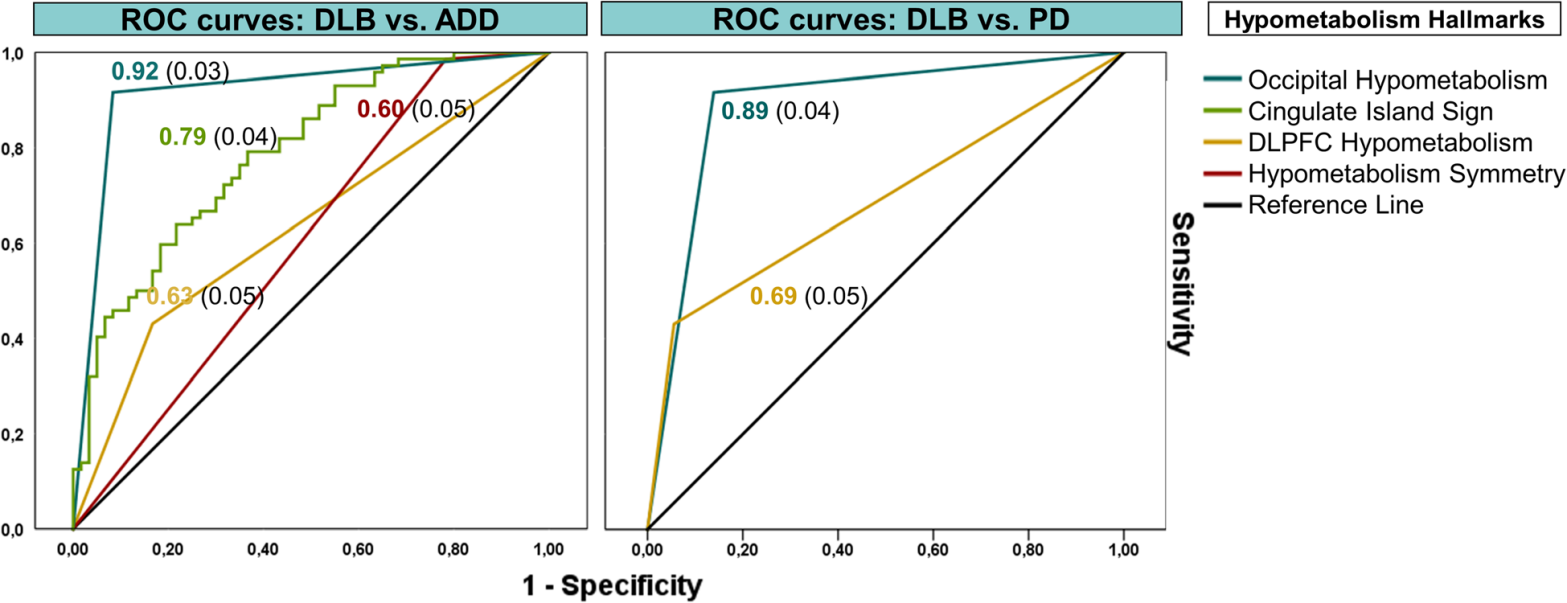
Received operating curves. Accuracy of the hypometabolism hallmarks obtained with the ROC analysis. The black line represents the diagonal reference. Abbreviations: ADD, Alzheimer’s disease dementia; DLB, dementia with Lewy bodies; PD, Parkinson’s disease; DLPFC, dorsolateral prefrontal cortex

The discriminative diagnostic performance of the SPM-t-map classification yielded an overall high accuracy: 0.92 (95%CI 0.86–0.96) accuracy in DLB vs. ADD classification and 0.95 (95% CI 0.89–0.98) accuracy in DLB vs. PD classification (Table 3). As for the candidate features, occipital hypometabolism had the highest discriminative ability in both the DLB vs. ADD and DLB vs. PD comparison. In discriminating DLB from ADD, the presence of occipital hypometabolism yielded significantly higher AUC than the presence of the cingulate island sign (*Z* = 3.01, *p* = 0.003) (Table 4; Fig. 4). The ROC delivered a CIS value of 0.82 as the optimal cut-off score to discriminate between DLB and ADD patients. Cingulate island sign and hypometabolism symmetry were not evaluated for the PD case series, since the great majority of PD patients did not present a clear-cut hypometabolism pattern and/or hypometabolism in the posterior cingulate cortex, precuneus, and cuneus, making evaluation of these hallmarks inappropriate.

**Table 4:** Prevalence and performance of selected hypometabolism hallmarks in discriminating DLB from ADD and PD patients

Prevalence (%) of selected hypometabolism hallmarks				
	Occipital hypometabolism	Cingulate island sign	DLPFC hypometabolism	Hypometabolism symmetry
DLB	66/72 (91.7%)	57/72 (79.2%)	31/72 (43.1%)	71/72 (98.6%)
ADD	5/60 (8.3%)	22/60 (36.7%)	10/60 (16.7%)	47/60 (78.3%)
PD	5/36 (13.9%)	–	2/36 (5.56%)	–
Performance of selected hypometabolism hallmarks				
	DLB vs. ADD			
*Hypometabolism hallmarks*	Sensitivity	Specificity	Accuracy	
Occipital hypometabolism	0.92 (0.83–0.97)	0.92 (0.82–0.97)	0.92 (0.86–0.96)	
Cingulate island sign	0.79 (0.68–0.88)	0.63 (0.50–0.75)	0.72 (0.63–0.79)	
DLPFC hypometabolism	0.43 (0.31–0.55)	0.83 (0.71–0.92)	0.61 (0.53–0.70)	
Hypometabolism symmetry	0.99 (0.93–1)	0.22 (0.12–0.34)	0.64 (0.55–0.72)	
	DLB vs. PD			
*Hypometabolism hallmarks*	Sensitivity	Specificity	Accuracy	
Occipital hypometabolism	0.92 (0.83–0.97)	0.86 (0.70–0.95)	0.90 (0.83–0.95)	
Cingulate island sign	**–**	**–**	–	
DLPFC hypometabolism	0.43 (0.31–0.55)	*0.94* (0.81–0.99)	0.60 (0.50–0.69)	
Hypometabolism symmetry	**–**	**–**	**–**	

*Abbreviations*: *ADD* Alzheimer’s disease dementia, *DLB* dementia with Lewy bodies, *DLPFC* dorsolateral prefrontal cortex, *PD* Parkinson’s disease

**Fig. 5:**
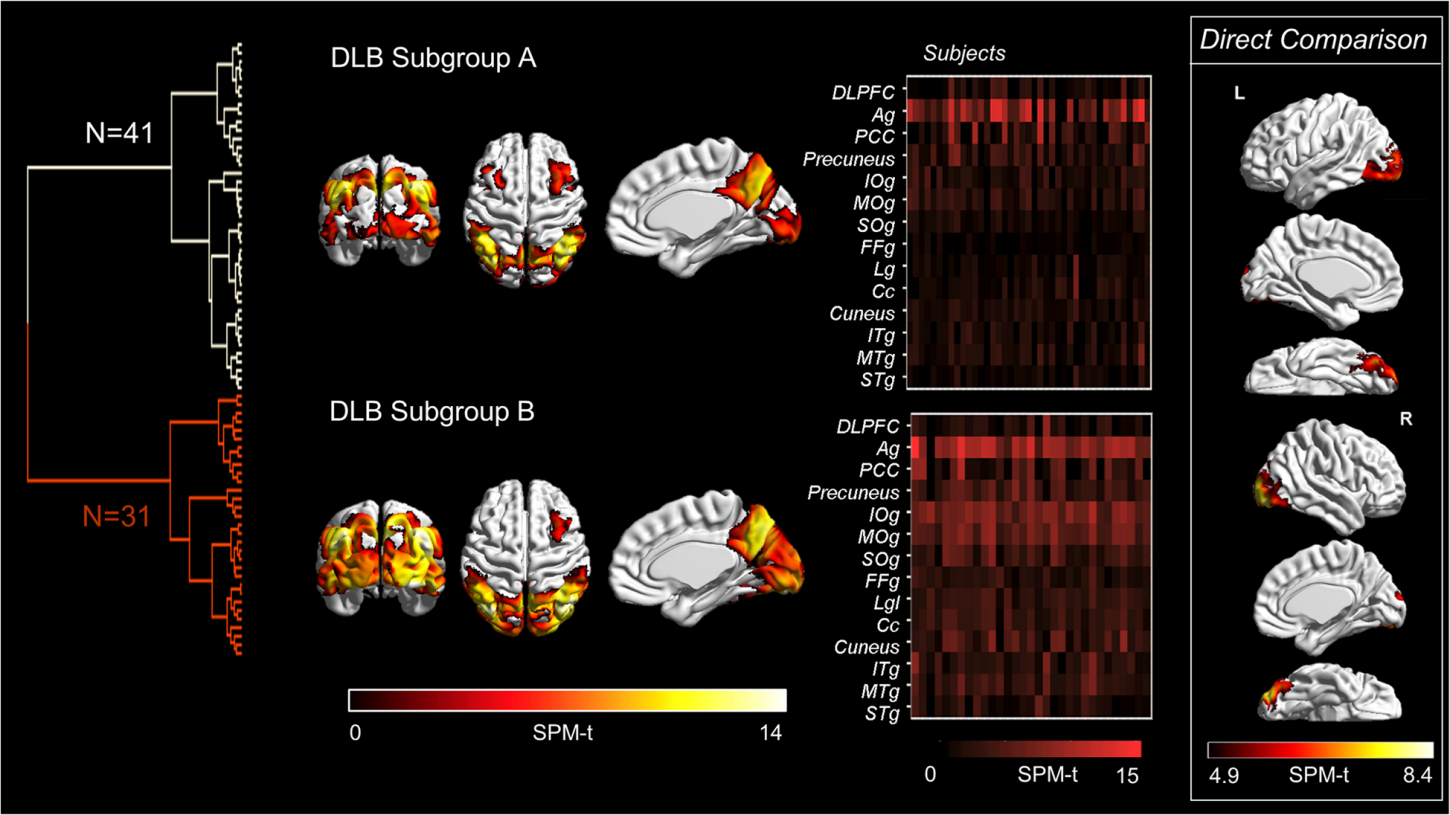
Hierarchical cluster analysis. Left panel, hypometabolism patterns differentiating the subgroup A (*N* = 41) and subgroup B (*N* = 31). Subgroups differed in severity of occipital hypometabolism, as shown in the brain renderings. Middle panel, raster plots showing the severity of regional hypometabolism at the single-subject level according to SPM-t values; ROIs are plotted on the ordinate axis; subjects on the abscissa axis. Right panel shows results of the head-to-head metabolic comparison between subgroups (*p* < 0.05 FWE-corrected, *k* = 100). Abbreviations: Ag, angular gyrus; Cc, calcarine cortex; DLPFC, dorsolateral prefrontal cortex; FFg, fusiform gyrus; IOg, inferior occipital gyrus; ITg, inferior temporal gyrus; Lg, lingual gyrus; MOg, middle occipital gyrus; MTg, middle temporal gyrus; SOg, superior occipital gyrus; STg, superior temporal gyrus

The *hierarchical cluster analysis* identified two DLB subgroups with a fairly comparable sample size (*N* = 41 and 31) and slightly different occipital hypometabolism (Fig. 5). The two DLB subgroups significantly differed both in averaged scores of cognitive performance, regardless of disease duration. More severe occipital involvement was significantly associated with worse global cognitive function, visuo-spatial, visuo-perceptive, and executive deficits (Table 2). Although symptom prevalence did not differ between clinical groups at baseline (Table 2), significant differences became evident at follow-up, with more severe occipital involvement associated to an extremely high prevalence of VH (93.5% vs. 63.4%; *χ*^2^ = 8.887, *p* < 0.005). More severe occipital involvement was also associated with a higher likelihood of *developing* VH: among VH-free patients at baseline, 84.6% of those with more severe occipital involvement developed VH at follow-up, as compared to only 34.8% of those with less severe occipital involvement (*χ*^2^ = 8.276, *p* < 0.005).

## Discussion

Previous [18F]FDG-PET studies in DLB mostly focused either on the assessment of [18F]FDG-PET hypometabolism patterns at the group level, thus not allowing for measures of diagnostic accuracy, or on the relationship between focal metabolic alterations and clinical symptoms.

In this study, we assessed the diagnostic accuracy of the disease-specific brain metabolic signatures at the single-subject level in a large series of patients with dementia with Lewy bodies. The results indicate a significant and consistent occipital hypometabolism pattern, as shown by the SPM-t-maps at the single-subject level, as a supportive and predictive hallmark for the diagnosis of DLB and, of note, for cases with uncertain diagnoses (atypical parkinsonisms or MCI) (see Fig. 1).

### [18F]FDG-PET SPM hypometabolism pattern in DLB

The [18F]FDG-PET SPM maps confirmed the presence of a specific hypometabolism pattern, both at the group and single-subject level (Fig. 2; Fig. 3), in our DLB case series. This hypometabolism pattern was characterized by occipital, parieto-temporal, and frontal involvement, consistent with previous reports on very limited case series [6, 23, 29, 33].

Notably, the cluster analysis revealed two hypometabolism subtypes (Fig. 5), with the severity of occipital hypometabolism driving most of the difference. The reason why brain occipital regions may show different degrees of metabolic involvement in DLB is unclear. Brain glucose hypometabolism patterns, as revealed by [18F]FDG-PET, are related to the underlying synaptic dysfunction, as determined by local and long-distance effects due to underlying neuropathology [34, 35]. The occipital hypometabolism found in DLB is independent of brain atrophy, as gross structural changes in the occipital lobe do not occur in patients with DLB [36].

In our series, more severe occipital involvement (Fig. 5) was predictive of a higher prevalence and higher likelihood of developing VH at follow-up, in keeping with the previously reported association between VH and occipital hypometabolism [37–39] and in support of the predictive ability of hypometabolism with respect to the development of clinical features. Deficits in the visuo-spatial, visuo-attention, and visuo-constructive cognitive domains, which have been suggested to promote VH [40–42], were also more severe in the subgroup with a more extensive occipital involvement. Converging evidence shows that both VH and neuropsychological deficits might be ascribed to cholinergic dysfunction [43–45]. The clinical and neuropsychological differences may therefore indicate different degrees of underlying cholinergic impairment, ultimately reflecting the variable occipital hypometabolism. It may also be speculated that the observed differences in severity of occipital hypometabolism could indicate the presence of a variable amount of neocortical occipital Lewy body pathology, supporting previous claims of possible DLB pathological subtypes [46, 47]. Accordingly, it has been suggested that Lewy body pathology could be the main determinant of occipital hypometabolism, whereas temporo-parietal hypometabolism in DLB might be driven by underlying AD pathology [48]. Consistently, and in keeping with our findings, pure Lewy body pathology has been associated with greater impairment in visuospatial and visual perceptive abilities [49, 50], whereas comorbid AD pathology was related to worse performance in other cognitive domains, such as memory and orientation [51].

### [18F]FDG-PET SPM hypometabolism as a biomarker for DLB

Here, in a large series of cases, we have shown that [18F]FDG-PET alone, when combined with optimized semi-quantitative approaches at the single-subject level, is able to *predict the diagnosis* of DLB 3 years before the final clinical diagnosis, with extremely high accuracy (≈ 90%). This provides insight into the sensitivity of [18F]FDG-PET to define pathological changes when clinical diagnosis alone is still uncertain. Considering the high clinical variability [1] and lack of a clear clinical picture in the initial phases of DLB, an early diagnosis based on clinical data alone may be challenging. Consistently, as shown in Table 1, at entry (i.e., clinical classification at entry), 10 out of 72 patients were classified as possible DLB, and 34 out of 72 as probable DLB. The remaining clinical presentations were attributed to other dementias, such as ADD, or classified as atypical parkinsonisms or MCI. The possible diagnostic misclassifications at entry may be partially due to the clinicians’ uncertainty about patients’ clinical presentations. Several factors may have influenced the low level of confidence for the presence of core symptoms, e.g., very doubtful presence of visual hallucinations and cognitive fluctuations, only reported by the relatives and not evaluated by standard sequential neuropsychological exams. The high power of the [18F]FDG-PET hypometabolism signatures in predicting the final clinical diagnosis provided a ≈ 50% increase in accuracy compared to the first clinical assessment alone (clinical classification at entry). In cases of diagnostic uncertainty, the use of the [18F]FDG-PET biomarker in the flowchart may help to specify the clinical diagnosis, predicting the clinical scenario years in advance (Fig. 1). It is important to note that [18F]FDG-PET raters were blinded to clinical diagnoses at entry and follow-up, in accordance with the main objective of the study. In reality, however, the final clinical diagnosis was obtained with, and reinforced by, available core clinical features and supportive biomarkers, consistent with consensus guidelines [5].

As for the differential diagnosis, the misdiagnosis of DLB as ADD is usually due to the overlap of both cognitive and behavioral symptoms [4]. Previous studies aimed at assessing [18F]FDG-PET as a biomarker for the *differential diagnosis* between DLB and ADD reported overall good, although not always optimal, accuracy values (cfr. [4]). When considering all available studies (see Additional file 1: Table S1 for an overview) with a fair sample size (*N* = 30) [52, 53], simple visual assessment produced accuracy values of around 70% (range 69–72%) and quantitative metrics produced an accuracy of 85%. Here, we adopted an optimized semi-quantitative SPM-based approach, which has previously shown excellent performance in discriminating dementia conditions [6, 54], outperforming visual qualitative assessment [6]. Our results indicate that occipital lobe hypometabolism, found here in 91.7% of DLB patients and yielding the greatest ability to discriminate between DLB and ADD, is the dysfunctional signature of DLB (Table 4; Fig. 4). The substantial, although suboptimal, accuracy produced using the cingulate island sign (ROC < 80%) in comparison with the other hallmarks confirms its value in supporting differential diagnosis with AD, as acknowledged in the new DLB diagnostic criteria [55]. Our cut-off CIS value of 0.82 to discriminate between DLB and ADD patients was consistent with a previous study showing that a CIS value of 0.81 optimally discriminated between AD and DLB pathology at post-mortem evaluation [27]. Additionally, another study found a CIS value of 0.79 as the optimal cut-off to discriminate between clinically diagnosed DLB and ADD patients [27]. It has been shown that a higher cingulate island sign value could be specifically associated with a lower neurofibrillary tangle (NFT) pathology Braak stage rather than with the presence of significant amyloid plaque deposition [56]. Our results are consistent with this claim, suggesting that most of our clinically diagnosed DLB patients were likely to have lower NFT pathology as compared to the ADD sample.

Notably, the DLB vs. ADD classification was hampered by the presence of 8.3% of cases with the PCA variant in the ADD sample. The differentiation between PCA and DLB hypometabolism patterns can be challenging due to the topographical overlap in the hypometabolism patterns between the two conditions [28] (Fig. 3). However, as shown in a recent study by Cerami et al., the focal hypometabolism of the frontal eye field regions may help in defining the PCA condition [25], while a more diffuse involvement of DLPFC is frequently reported in DLB and not in PCA (Fig. 3).

Difficulty with diagnostic differentiation between DLB and atypical parkinsonisms is also frequent, due to ambiguous clinical presentations characterizing the initial clinical stages [57]. Hughes et al. reported that although the clinical diagnosis at follow-up reached an 85% accuracy in predicting the post-mortem diagnosis, the clinical classification at entry was revised in 60% of patients [58]. When the clinical scenario is still ambiguous, [18F]FDG-PET can provide patterns of brain hypometabolism able to accurately discriminate between DLB, progressive supranuclear palsy, cortico-basal degeneration, and multiple system atrophy [23]. In a previous study, the [18F]FDG-PET single-subject procedure was applied in a more limited DLB case series (*N* = 29), showing promising discriminative abilities with respect to other atypical parkinsonian disorders in the early diagnostic phase [23].

As for the DLB vs. PD classification, it must be noted that most of the PD patients (84%) presented with the typical PD-like [18F]FDG-PET pattern [24] (excellently discriminated from DLB by the SPM-t-map classification). These stable, cognitively normal patients with PD might present with executive-attention deficits [59] and very limited prefrontal hypometabolism, contrasting with the prevalent posterior cortical dysfunction characterizing DLB.

On the other hand, a small number (*N* = 5, 13.8%) of PD cases exhibited the pattern typically found in DLB, characterized by occipital and posterior parietal-temporal hypometabolism (Table 3). The DLB-like pattern has been previously observed in patients with Parkinson’s disease [60, 61]. Crucially, the DLB-like pattern was detected at the individual level in PD patients, even in the early disease phase, and was associated with an increased risk of developing dementia along the disease course [24]. The hypometabolism overlap in patients on the trajectory to Parkinson’s disease with dementia (PDD) and those with DLB is not surprising, considering the phenotypical, genetic, and pathological similarities between the two syndromes [62, 63]. Accordingly, the PDD/DLB distinction is today considered essentially a clinical one and purely based on the chronology of symptom presentation. The use of [18F]FDG-PET hypometabolism t-maps is thus critical in distinguishing between DLB/PDD and stable PD and is of extreme importance for prognostic accuracy and treatment options.

[18F]FDG-PET hypometabolism represents a flexible biomarker, able to differentiate DLB not only from ADD, but also from other parkinsonisms, either typical (as shown here) or atypical (see [23]). This is different from DaTSCAN, currently included as an indicative biomarker in the DLB diagnostic criteria, which only provides a dichotomous discrimination between parkinsonisms and ADD and does not allow for a more fine-grained differentiation between DLB and typical and atypical parkinsonisms [4, 23, 55]. Notably, [18F]FDG-PET, combined with semi-quantitative methods, allows for discrimination between DLB and ADD with accuracy even superior to that of DaTSCAN, whose sensitivity (78%), is also hampered by the negligible dopaminergic neurodegeneration characterizing some DLB cases (cfr. [4]).

The [18F]FDG-PET classification showed substantial inter-rater agreement, consistent with previous validation studies in other neurodegenerative conditions [6, 23, 54, 64, 65]. This supports the reliability of the [18F]FDG-PET SPM maps for diagnostic classification, suggesting that the assessment of disease-specific hypometabolism patterns obtained by this [18F]FDG-PET SPM procedure is not influenced by the specific rater expertise. Our SPM-t-maps, derived from voxel-by-voxel comparisons between an individual subject and a predefined normal database, provide fast and easily accessible information about the distribution of brain hypometabolism that does not require rater expertise. Similarly, a previous comparative study showed that the SPM-t-maps of brain hypometabolism provide the clinician with a higher degree of accuracy compared to visualization of standard rainbow maps of FDG uptake [6]. The effectiveness of this procedure in clinical routine is supported by the independence of clinicians’ expertise, as shown by comparable results among raters.

### Limitations

This is a retrospective study, in which patients’ data were obtained by records collected during clinical routine, thus without following a standardized procedure. In some cases, the presence of the core symptoms (VH and cognitive fluctuations) was based on the caregivers’ reports only, without further specific testing. This might have produced some clinical misclassifications at entry. It is to note that the classification of possible or probable DLB at baseline does not affect the final results on statistical concordance, since both possible and probable DLB diagnoses were equally referred to the DLB category. Finally, [18F]FDG-PET was available, together with other biomarkers, to support the final diagnosis, and this may have influenced the clinicians’ final decision. Even if this study findings support the informative role of [18F]FDG-PET in the very early clinical phase of the diagnostic flowchart, replications considering post-mortem pathological diagnosis are needed.

## Conclusions

We conclude that the typical brain hypometabolism pattern, crucially involving the occipital lobes and obtained with appropriate analytic methods, represents a reliable biomarker of DLB, thus supporting its inclusion in the current diagnostic criteria. This evidence also advocates for a reconsideration of the diagnostic flowchart for DLB and promotes the use of [18F]FDG-PET as a valuable biomarker that is very accurate in early DLB diagnosis and in the differential diagnosis with other parkinsonian and dementia conditions. Considering the high uncertainty of the first clinical assessment in parkinsonian and DLB conditions, [18F]FDG-PET, combined with optimized semi-quantitative approaches, is an effective tool to support differential diagnosis when clinical assessment is not yet reliably informative, thus shortening the diagnostic process. An accurate identification of the DLB condition from the first clinical assessment is critical in planning necessary therapeutic, organizational, and rehabilitative interventions.

## Additional file

Additional file 1: Table S1. Summary of [18F]FDG-PET accuracy studies in DLB (DOCX 21 kb)

## Abbreviations

AD: Alzheimer’s disease
ADD: Alzheimer’s disease dementia
DLB: Dementia with Lewy bodies
DLPFC: Dorsolateral prefrontal cortex
MCI: Mild cognitive impairment
PCA: Posterior cortical atrophy
PD: Parkinson’s disease
PDD: Parkinson’s disease with dementia
SPM: Statistical Parametric Mapping
VH: Visual hallucinations

## References

[1] Rizzo G, Arcuti S, Copetti M, Alessandria M, Savica R, Fontana A (2018). Accuracy of clinical diagnosis of dementia with Lewy bodies: a systematic review and meta-analysis. J Neurol Neurosurg Psychiatry.

[2] Kato T, Inui Y, Nakamura A, Ito K (2016). Brain fluorodeoxyglucose (FDG) PET in dementia. Ageing Res Rev.

[3] Bohnen NI, Djang DSW, Herholz K, Anzai Y, Minoshima S (2012). Effectiveness and safety of 18F-FDG PET in the evaluation of dementia: a review of the recent literature. J Nucl Med.

[4] McKeith I, Boeve B, Dickson D, Lowe J, Emre M (2017). Diagnosis and management of dementia with Lewy bodies: fourth consensus report of the DLB consortium. Neurol.

[5] McKeith IG, Dickson DW, Lowe J, Emre M, O’Brien JT, Feldman H (2005). Diagnosis and management of dementia with Lewy bodies: third report of the DLB consortium. Neurol.

[6] Perani D, Della Rosa PA, Cerami C, Gallivanone F, Fallanca F, Vanoli EG (2014). Validation of an optimized SPM procedure for FDG-PET in dementia diagnosis in a clinical setting. NeuroImage Clin.

[7] Perani D, Orazio S, Padovani A, Nobili FM, Iaccarino L, Della Rosa PA (2014). A survey of FDG- and amyloid-PET imaging in dementia and grade analysis. Biomed Res Int.

[8] Frisoni GB, Bocchetta M, Chételat G, Rabinovici GD, De Leon MJ, Kaye J (2013). Imaging markers for Alzheimer disease: which vs how. Neurol.

[9] Caroli A, Prestia A, Chen K, Ayutyanont N, Landau SM, Madison CM (2012). Summary metrics to assess Alzheimer disease-related hypometabolic pattern with 18F-FDG PET: head-to-head comparison. J Nucl Med.

[10] Frisoni GB, Perani D, Bastianello S, Bernardi G, Cappa SF, Trabucchi M (2017). A roadmap to the use of biomarkers for the diagnosis of Alzheimer’s disease in clinical practice: the Italian inter-societal consensus. Alzheimers Dement.

[11] Garibotto V, Herholz K, Boccardi M, Picco A, Varrone A, Nordberg A (2017). Clinical validity of brain fluorodeoxyglucose positron emission tomography as a biomarker for Alzheimer’s disease in the context of a structured 5-phase development framework. Neurobiol Aging.

[12] Guerra UP, Nobili FM, Padovani A, Perani D, Pupi A, Sorbi S (2015). Recommendations from the Italian Interdisciplinary Working Group (AIMN, AIP, SINDEM) for the utilization of amyloid imaging in clinical practice. Neurol Sci.

[13] Albert MS, DeKosky ST, Dickson D, Dubois B, Feldman HH, Fox NC (2011). The diagnosis of mild cognitive impairment due to Alzheimer’s disease: recommendations from the National Institute on Aging and Alzheimer’s association workgroup. Alzheimers Dement.

[14] Cummings JL, Mega M, Gray K, Rosenberg-Thompson S, Carusi DA, Gornbein J (1994). The neuropsychiatric inventory: comprehensive assessment of psychopathology in dementia. Neurol.

[15] Lee DR, McKeith I, Mosimann U, Ghosh-Nodial A, Grayson L, Wilson B (2014). The dementia cognitive fluctuation scale, a new psychometric test for clinicians to identify cognitive fluctuations in people with dementia. Am J Geriatr Psychiatry.

[16] McKhann GM, Knopman DS, Chertkow H, Hyman BT, Jack CR, Kawas CH (2011). The diagnosis of dementia due to Alzheimer’s disease: recommendations from the National Institute on Aging-Alzheimer’s Association workgroups on diagnostic guidelines for Alzheimer’s disease. Alzheimers Dement.

[17] Snowden JS, Stopford CL, Julien CL, Thompson JC, Davidson Y, Gibbons L (2007). Cognitive phenotypes in Alzheimer’s disease and genetic risk. Cortex..

[18] Hughes AJ, Daniel SE, Kilford L, Lees AJ (1992). Accuracy of clinical diagnosis of idiopathic Parkinson’s disease: a clinico-pathological study of 100 cases. J Neurol Neurosurg Psychiatry.

[19] Varrone A, Asenbaum S, Vander Borght T, Booij J, Nobili F, Nagren K (2009). EANM procedure guidelines for PET brain imaging using [18F]FDG, version 2. Eur J Nucl Med Mol Imaging.

[20] Signorini M, Paulesu E, Friston K, Perani D, Colleluori A, Lucignani G (1999). Rapid assessment of regional cerebral metabolic abnormalities in single subjects with quantitative and nonquantitative [18F]FDG PET: a clinical validation of statistical parametric mapping. Neuroimage..

[21] Gallivanone F, Della Rosa P, Perani D, Gilardi MC, Castiglioni I (2017). The impact of different 18FDG PET Healthy Subject scans for comparison with single patient in SPM analysis. Q J Nucl Med Mol imaging.

[22] Della Rosa PA, Cerami C, Gallivanone F, Prestia A, Caroli A, Castiglioni I (2014). A standardized [18F]-FDG-PET template for spatial normalization in statistical parametric mapping of dementia. Neuroinformatics.

[23] Caminiti SP, Alongi P, Majno L, Volontè MA, Cerami C, Gianolli L (2017). Evaluation of an optimized [18F]fluoro-deoxy-glucose positron emission tomography voxel-wise method to early support differential diagnosis in atypical parkinsonian disorders. Eur J Neurol.

[24] Pilotto A, Premi E, Caminiti SP, Presotto L, Alberici A, Paghera B (2018). Single-subject SPM FDG-PET patterns predict risk of dementia progression in Parkinson’s disease. Neurol.

[25] Cerami C, Crespi C, Della Rosa PA, Dodich A, Marcone A, Magnani G (2015). Brain changes within the visuo-spatial attentional network in posterior cortical atrophy. J Alzheimers Dis.

[26] Pappatà S, Santangelo G, Aarsland D, Vicidomini C, Longo K, Bronnick K (2011). Mild cognitive impairment in drug-naive patients with PD is associated with cerebral hypometabolism. Neurol.

[27] Lim SM, Katsifis A, Villemagne VL, Best R, Jones G, Saling M (2009). The 18F-FDG PET cingulate island sign and comparison to 123I-beta-CIT SPECT for diagnosis of dementia with Lewy bodies. J Nucl Med.

[28] Whitwell JL, Graff-Radford J, Singh TD, Drubach DA, Senjem ML, Spychalla AJ (2017). 18 F-FDG PET in posterior cortical atrophy and dementia with Lewy bodies. J Nucl Med.

[29] Teune LK, Bartels AL, de Jong BM, ATM W, Eshuis SA, de Vries JJ (2010). Typical cerebral metabolic patterns in neurodegenerative brain diseases. Mov Disord.

[30] Tzourio-Mazoyer N, Landeau B, Papathanassiou D, Crivello F, Etard O, Delcroix N (2002). Automated anatomical labeling of activations in SPM using a macroscopic anatomical parcellation of the MNI MRI single-subject brain. Neuroimage.

[31] R Development Core Team (2013). R: A language and environment for statistical computing.

[32] Lê S, Josse J, Husson F (2008). FactoMineR: an R package for multivariate analysis. J Stat Softw.

[33] Cerami C, Dodich A, Greco L, Iannaccone S, Magnani G, Marcone A (2016). The role of single-subject brain metabolic patterns in the early differential diagnosis of primary progressive aphasias and in prediction of progression to dementia. J Alzheimers Dis.

[34] Perani D (2014). FDG-PET and amyloid-PET imaging: the diverging paths. Curr Opin Neurol.

[35] Higuchi M, Tashiro M, Arai H, Okamura N, Hara S, Higuchi S (2000). Glucose hypometabolism and neuropathological correlates in brains of dementia with Lewy bodies. Exp Neurol.

[36] Middelkoop HAM, van Der Flier WM, Burton EJ, Lloyd AJ, Paling S, Barber R (2001). Dementia with Lewy bodies and AD are not associated with occipital lobe atrophy on MRI. Neurol.

[37] Imamura T, Ishii K, Hirono N, Hashimoto M, Tanimukai S, Kazuai H (1999). Visual hallucinations and regional cerebral metabolism in dementia with Lewy bodies (DLB). Neuroreport..

[38] Firbank MJ, Lloyd J, O’Brien JT (2016). The relationship between hallucinations and FDG-PET in dementia with Lewy bodies. Brain Imaging Behav.

[39] Perneczky R, Drzezga A, Boecker H, Förstl H, Kurz A, Häussermann P (2008). Cerebral metabolic dysfunction in patients with dementia with Lewy bodies and visual hallucinations. Dement Geriatr Cogn Disord.

[40] Cagnin A, Gnoato F, Jelcic N, Favaretto S, Zarantonello G, Ermani M (2013). Clinical and cognitive correlates of visual hallucinations in dementia with Lewy bodies. J Neurol Neurosurg Psychiatry.

[41] Collerton D, Perry E, McKeith I (2005). Why people see things that are not there: a novel Perception and Attention Deficit model for recurrent complex visual hallucinations. Behav Brain Sci.

[42] Hamilton JM, Salmon DP, Galasko D, Raman R, Emond J, Hansen LA (2008). Visuospatial deficits predict rate of cognitive decline in autopsy-verified dementia with Lewy bodies. Neuropsychol.

[43] Mori T, Ikeda M, Fukuhara R, Nestor PJ, Tanabe H (2006). Correlation of visual hallucinations with occipital rCBF changes by donepezil in DLB. Neurology.

[44] Dotigny F, Ben Amor AY, Burke M, Vaucher E (2008). Neuromodulatory role of acetylcholine in visually-induced cortical activation: behavioral and neuroanatomical correlates. Neuroscience.

[45] Satoh M, Ishikawa H, Meguro K, Kasuya M, Ishii H, Yamaguchi S (2010). Improved visual hallucination by donepezil and occipital glucose metabolism in dementia with lewy bodies: the Osaki-Tajiri project. Eur Neurol.

[46] Dugger BN, Boeve BF, Murray ME, Parisi JE, Fujishiro H, Dickson DW (2012). Rapid eye movement sleep behavior disorder and subtypes in autopsy-confirmed dementia with Lewy bodies. Mov Disord.

[47] Toledo JB, Gopal P, Raible K, Irwin DJ, Brettschneider J, Sedor S (2016). Pathological α-synuclein distribution in subjects with coincident Alzheimer’s and Lewy body pathology. Acta Neuropathol.

[48] Kasanuki K, Iseki E, Fujishiro H, Yamamoto R, Higashi S, Minegishi M (2012). Neuropathological investigation of the hypometabolic regions on positron emission tomography with [18F] fluorodeoxyglucose in patients with dementia with Lewy bodies. J Neurol Sci.

[49] Yoshizawa H, Vonsattel JPG, Honig LS (2013). Early neuropsychological discriminants for Lewy body disease: an autopsy series. J Neurol Neurosurg Psychiatry.

[50] Peavy GM, Edland SD, Toole BM, Hansen LA, Galasko DR, Mayo AM (2016). Phenotypic differences based on staging of Alzheimer’s neuropathology in autopsy-confirmed dementia with Lewy bodies. Park Relat Disord.

[51] Andersson M, Zetterberg H, Minthon L, Blennow K, Londos E (2011). The cognitive profile and CSF biomarkers in dementia with Lewy bodies and Parkinson’s disease dementia. Int J Geriatr Psychiatry.

[52] O’Brien JT, Firbank MJ, Davison C, Barnett N, Bamford C, Donaldson C (2014). 18F-FDG PET and perfusion SPECT in the diagnosis of Alzheimer and Lewy body dementias. J Nucl Med.

[53] Firbank MJ, Lloyd J, Williams D, Barber R, Colloby SJ, Barnett N (2016). An evidence-based algorithm for the utility of FDG-PET for diagnosing Alzheimer’s disease according to presence of medial temporal lobe atrophy. Br J Psychiatry.

[54] Perani D, Cerami C, Caminiti SP, Santangelo R, Coppi E, Ferrari L (2016). Cross-validation of biomarkers for the early differential diagnosis and prognosis of dementia in a clinical setting. Eur J Nucl Med Mol Imaging.

[55] McKeith I, Dickson D, Lowe J, Emre M (2017). Diagnosis and management of dementia with Lewy bodies. Neurol.

[56] Graff-Radford J, Murray ME, Lowe VJ, Boeve BF, Ferman TJ, Przybelski SA (2014). Dementia with Lewy bodies: basis of cingulate island sign. Neurol.

[57] Joutsa J, Gardberg M, Röyttä M, Kaasinen V (2014). Diagnostic accuracy of parkinsonism syndromes by general neurologists. Park Relat Disord.

[58] Hughes AJ, Daniel SE, Ben-Shlomo Y, Lees AJ (2002). The accuracy of diagnosis of parkinsonian syndromes in a specialist movement disorder service. Brain..

[59] Kehagia AA, Barker RA, Robbins TW (2012). Cognitive impairment in Parkinson’s disease: the dual syndrome hypothesis. Neurodegener Dis.

[60] Bohnen NI, Koeppe RA, Minoshima S, Giordani B, Albin RL, Frey KA (2011). Cerebral glucose metabolic features of Parkinson disease and incident dementia: longitudinal study. J Nucl Med.

[61] Baba T, Hosokai Y, Nishio Y, Kikuchi A, Hirayama K, Suzuki K (2017). Longitudinal study of cognitive and cerebral metabolic changes in Parkinson’s disease. J Neurol Sci.

[62] McKeith IG (2018). Author response: diagnosis and management of dementia with Lewy bodies: fourth consensus report of the DLB consortium. Neurol.

[63] Friedman JH (2018). Dementia with Lewy bodies and Parkinson disease dementia: it is the same disease!. Park Relat Disord.

[64] Cerami C, Della Rosa PA, Magnani G, Santangelo R, Marcone A, Cappa SF (2015). Brain metabolic maps in mild cognitive impairment predict heterogeneity of progression to dementia. NeuroImage Clin.

[65] Iaccarino L, Chiotis K, Alongi P, Almkvist O, Wall A, Cerami C, et al. A cross-validation of FDG- and amyloid-PET biomarkers in mild cognitive impairment for the risk prediction to dementia due to Alzheimer’s disease in a clinical setting. J Alzheimers Dis. 2017;59(2):603–14. 10.3233/JAD-170158 28671117

