# Supplementary material for: Brain glucose metabolism in Lewy body dementia: implications for diagnostic criteria
Source: Alzheimers Res Ther. 2019 Feb 23;11:20. doi: 10.1186/s13195-019-0473-4 (PMC6387558; doi:10.1186/s13195-019-0473-4)
Supplement: Supplementary file 1 — Table S1. Summary of [18F]FDG-PET accuracy studies in DLB (DOCX 21 kb) [file 13195_2019_473_MOESM1_ESM.docx]

**Additional file 1: Table 1. Summary of [18F]FDG-PET accuracy studies in DLB**

| **Reference** | **Analysis** | **Sample Size (N)** | **Autopsy** | **Sensitivity/Specificity/Accuracy** |
| --- | --- | --- | --- | --- |
| Ishii et al., 1998 | Quantitative CMRglu | DLB : 12  ADD: 12 | Absent | **DLB vs. ADD**  ***Occipital CMRglu:*** .92/.92/.92 |
| Higuchi et al., 2000 | Z-scores | DLB: 7  ADD: 11 | LBD: 1 | **DLB vs .ADD**  ***Visual association cortex Z-score:***  .86/.91/.89 |
| Minoshima et al., 2001 | 3D-SSP | DLB: 11  ADD: 10 | pure LBD: 4  LBD+AD: 7  pure AD: 10 | **DLB vs. ADD**  ***Occipital lobe Z-score:***.90/.80/.86 |
| Koeppe et al., 2005 | SUVR (ROIs) | DLB: 20 ADD: 25 | Absent | **DLB vs. ADD**  ***BA24-BA17 SUVR:*** .85 ./84 /.84 |
| Gilman et al., 2005 | Quantitative CMRglc | DLB:20  ADD: 25 | Absent | **DLB. vs. ADD**  ***BA 17 CMRGlc:***.64/.65 /NA |
| Ishii et al., 2007 | SPM-analysis | DLB: 20  ADD: 20  HC: 20 | Absent | **DLB vs. ADD**  ***Occipital/pons ratio:*** NA/NA/.73  ***Occipital lobe/hippocampus ratio:***  NA/NA/.87 |
| Kono et al., 2007 | Visual assessment  Z-score | DLB: 16  ADD: 21 | Absent | **DLB vs. ADD**  ***Visual assessment:*** NA/NA/≈.60-.69  ***Automated diagnosis system:*** .75/.72/.73 |
| Mosconi et al., 2008 | Z-Score | DLB: 27  ADD: 199  FTD: 98 | Absent | ***Standardized automated analysis:***  **DLB vs. ADD:** .99 /.71 /.97  **DLB vs. FTD:** .71 /.65 /.68 |
| Lim et al., 2009 | Visual assessment;  SUVr (ROIs);  Visual assessment(aided by NEUROSTAT 3D-SSP) | DLB: 14  ADD: 10 | LBD+AD:3 pure LBD: 1  AD: 1 | **DLB vs. ADD**  ***Visual assessment:***  -*hypometabolism pattern:* 83 /93 /NA  -*medial occipital cortex*: 46/97/NA  -*lateral occipital cortex*: 88/80/NA  -*CIS:* .73/100/NA  ***ROI analysis:***  -*medial occipital cortex*: .77/.80/.78  -*lateral occipital cortex:* .77/.80/.78  -*CIS*: .77/.80/.78  ***Aided visual assessment:*** .73/.83/.75 |
| Kantarci et al., 2012 | SPM- analysis | DLB: 21  ADD: 21 | LBD+AD: 3  pure AD: 2 | **DLB vs. ADD**  ***Occipital lobe uptake:*** NA/NA/.84 (AUROC)  ***CIS:*** NA/NA/.92(AUROC) |
| O’Brien et al., 2014 | Visual assessment;  SUVR (ROIs) | DLB: 30  ADD: 38 | Absent | **DLB vs. ADD**  ***Visual assessment:*** .74/.70/.72  ***Medial occipital/medial temporal lobe ratio:*** NA/NA/.85(AUROC) |
| Perani et al, 2014 | Visual assessment;  SPM-analysis | DLB: 9  ADD: 27  FTLD: 24 | Absent | **Global comparison:**  ***Visual assessment:*** .96/.84/NA  ***Visual assessment aided by SPM***: .78/.50/NA |
| Cerami et al., 2015 | SPM-analysis | MCI: 45 (2 MCI due to DLB) | Absent | **Global comparison:**  An exact binomial sign test indicated that the [18F]FDG-PET significantly classified 79% of all MCI patients |
| Chiba et al., 2016 | Z-score (Extent Ratio in BA 17,18,19) | DLB: 9  ADD: 9  MCI due to DLB: 9  MCI due to ADD: 8 | Absent | **DLB vs. ADD**  ***BA17 Z-score:*** NA/NA/≈.80 (AUROC)  ***BA18 Z-score:*** NA/NA/.84 (AUROC)  ***BA19 Z-score:***NA/NA/≈.80 (AUROC)  **MCI-DLB vs. MCI-AD**  ***BA 17 Z-score:*** NA/NA/ ≈.86 (AUROC)  ***BA 18 Z-score:*** NA/NA/≈.88 (AUROC)  ***BA 19 Z-score:***NA/NA/≈.76 (AUROC) |
| Firbank et al., 2016 | Visual assessment;  SUVR (ROIs) | DLB: 30  ADD: 37 | Absent | **DLB vs. ADD:**  ***Visual assessment:*** 59/.80/.69  ***Occipital/MTL ratio:*** .89/.80/.85  **Low MTLA DLB vs. ADD**  ***Visual assessment:*** NA/NA/.83  ***Occipital/MTL ratio:*** NA/NA/.85  **High MTLA DLB vs. ADD**  ***Visual assessment:*** NA/NA/.69  ***Occipital/MTL ratio:*** NA/NA/.72 |
| Caminiti et al., 2017 | SPM-analysis | DLB: 29  PSP: 22  CBD: 11 MSA-c: 8 | Absent | **Global comparison**  ***Visual assessment aided by SPM:***  .98/.99/.99 |
| Whitwell et al., 2017 | Visual assessment;  SPM (ROIs) | DLB: 18  PCA: 16 | Absent | **DLB vs. PCA**  ***Visual assessment of CIS:***.77/.63/NA  ***SPM (ROIs):***  *Calcarine gyrus*: .79/.69/NA  *Lingual gyrus*: .79/.69/NA  *Temporal pole:* .92/.72/NA  *Orbitofrontal cortex*: .80/.73/NA  *Prefrontal cortex:* .89/.62/NA  *Caudate:* .85/.71/NA |

Abbreviations: AD: Alzheimer’s disease; ADD: Alzheimer’s disease dementia; AUROC: area under curve; BA: Brodmann area; CBD: Corticobasal degeneration; CIS: cingulate island sign; CMRglu: cerebral metabolic rate of glucose; DLB: Lewy body dementia; FTD: fronto-temporal dementia; FTLD: fronto-temporal lobar degeneration; LBD: Lewy Body disease; MCI: Mild cognitive impairment; MSA-c: Multiple system atrophy –cerebellar variant; MTLA: medial temporal lobe atrophy; PCA: Posterior cortical atrophy; PSP: Progressive supranuclear palsy; ROI: regions of interest; SPM: statistical parametric mapping; SUVr: standardized uptake value ratio.
